# Supplementary material for: Accuracy of deep learning-based computed tomography diagnostic system for COVID-19: A consecutive sampling external validation cohort study
Source: PLoS One. 2021 Nov 4;16(11):e0258760. doi: 10.1371/journal.pone.0258760 (PMC8568139; doi:10.1371/journal.pone.0258760)
Supplement: S2 Table — Model for Individual Prognosis or Diagnosis Statement. (DOCX) [file pone.0258760.s003.docx]

S2 Table. The list of institutions where patient medical data.

1. Hyogo Prefectural Amagasaki General Medical Center
2. Kobe City Medical Center General Hospital
3. St. Marianna University School of Medicine
4. Sakai City Medical Center
5. Kyoto City Hospital
6. Tokyobay Urayasu Ichikawa Medical Center
7. Kawasaki Municipal Kawasaki Hospital
8. Yamanashi Prefectural Central Hospital
9. Osaka City General Hospital
10. National Defence Medical College Hospital
11. Hyogo Prefectural Kakogawa Medical Center
