# Supplementary material for: Accuracy of deep learning-based computed tomography diagnostic system for COVID-19: A consecutive sampling external validation cohort study
Source: PLoS One. 2021 Nov 4;16(11):e0258760. doi: 10.1371/journal.pone.0258760 (PMC8568139; doi:10.1371/journal.pone.0258760)
Supplement: S3 Table — Patients who met the following criteria even for one item were considered symptomatic and were enrolled in the study. (DOCX) [file pone.0258760.s004.docx]

S3 Table. Inclusion criteria.

| **Symptom** | **Level** |
| --- | --- |
| Fever | ≥ 37.0 C |
| Systolic blood pressure | ≤ 90 mmHg |
| Heart rate | ≥ 120 bpm |
| Respiratory rate | ≥ 25 /min |
| SpO2 | ≤ 92% |
| Use of catecholamine | Yes |
| Use of oxygen support | Yes |

Spo2, oxygen saturation; bpm, beats per minute
