# Supplementary material for: Accuracy of deep learning-based computed tomography diagnostic system for COVID-19: A consecutive sampling external validation cohort study
Source: PLoS One. 2021 Nov 4;16(11):e0258760. doi: 10.1371/journal.pone.0258760 (PMC8568139; doi:10.1371/journal.pone.0258760)
Supplement: S4 Table. Computed tomography system and protocol — (DOCX) [file pone.0258760.s005.docx]

S4 Table. Computed tomography system and protocol.

| Facility | C01 | C02 | C03 | C04 | C05 | C06 | C07 | C08 | | C09 | C10 | C11 |
| --- | --- | --- | --- | --- | --- | --- | --- | --- | --- | --- | --- | --- |
| System | Aquilion PRIME | Optima CT660 | Aquilion PRIME | Optima CT660 | Optima CT660 | Aquilion PRIME | Aquilion CX Edition | Aquilion ONE | Aquilion CXL | Aquilion PRIME | Aquilion CXL | Aquilion CX Edition |
| Vendor | Canon Medical Systems | GE | Canon Medical Systems | GE | GE | Canon Medical Systems | Canon Medical Systems | Canon Medical Systems | Canon Medical Systems | Canon Medical Systems | Canon Medical Systems | Canon Medical Systems |
| Tube voltage (kVp) | 120 | 120 | 120 | 120 | 120 | 120 | 120 | 120 | 120 | 120 | 120 | 120 |
| Automatic tube current Modulation (mAs) | Auto | 100-510 | 150-250 | 80-500 | 80-500 | 150-250 | 403-500 | 100-400 | 100-400 | 50-250 | 100-400 | 100-400 |
| Pitch |  |  |  |  |  |  |  |  |  |  |  |  |
| Standard | 111 | 55 | 65 | 55 | 55 | 65 |  | 65 | 53 | 65 | 53 |  |
| Factor | 0.813 | 0.984 | 0.813 | 0.984 | 0.984 | 0.813 | 1.172 | 0.813 | 0.828 | 0.813 | 0.828 | 1 |
| Matrix | 512 × 512 | 512 × 512 | 256 × 256 | 512 × 512 | 512 × 512 | 512 ×512 | 512 ×512 | 512 × 512 | 512 × 512 | 512 × 512 | 512 × 512 | 512 × 512 |
| Slice thickness | 0.5 × 80 | 0.625 × 64 | 0.5 × 80 | 0.625 × 64 | 0.625 × 64 | 0.5 × 80 | 0.5 × 64 | 0.5 × 80 | 0.5 × 64 | 0.5 × 80 | 1.0 × 64 | 5.0 × 64 |
| Field of view (mm) | 320 | 340 | 320 |  |  | 330 | 350 | 320 | 320 | 320-400 | 320 | 320 |
| Reconstruction interval (mm) | 5 | 0.625 | 2 | 1.25 | 1.25 | 3 | 5 | 5 | 5 | 5 | 5 | 5 |
