# Supplementary material for: Accuracy of deep learning-based computed tomography diagnostic system for COVID-19: A consecutive sampling external validation cohort study
Source: PLoS One. 2021 Nov 4;16(11):e0258760. doi: 10.1371/journal.pone.0258760 (PMC8568139; doi:10.1371/journal.pone.0258760)
Supplement: S5 Table — (DOCX) [file pone.0258760.s006.docx]

S5 Table. Population characteristics in development from datasheet

| Category | | Number (%) | |
| --- | --- | --- | --- |
| ***Diagnosis*** | COVID-19 | 3,722 | (42.9) |
|  | Pneumonia | 2,491 | (28.7) |
|  | Non-pneumonia | 2,454 | (28.3) |
| ***Sex*** | Male | 4,162 | (48.0) |
|  | Female | 3,826 | (44.1) |
| ***Age*** | ≤50 years | 3,137 | (36.1) |
|  | > 50 years | 4,851 | (55.9) |

COVID-19, coronavirus disease
