# Supplementary material for: Accuracy of deep learning-based computed tomography diagnostic system for COVID-19: A consecutive sampling external validation cohort study
Source: PLoS One. 2021 Nov 4;16(11):e0258760. doi: 10.1371/journal.pone.0258760 (PMC8568139; doi:10.1371/journal.pone.0258760)
Supplement: S1 File — (PDF) [file pone.0258760.s007.pdf]

Laboratory Report

Title

- **COVID-19 AI Assisted Analysis Based On Chest CT Imaging**

Introduction & Background

COVID-19 has widely spread all over the world since the beginning of 2020. It is highly contagious and may lead to acute respiratory distress or multiple organ failure in severe cases.

- **Background**

  The Reverse-Transcription Polymerase Chain Reaction (RT-PCR) has been regards as the major reference for COVID-19 diagnoses. However, it has been reported that the sensitivity of RT-PCR might not be high enough for the purpose of early detection and treatment of the presumptive patients. Several studies indicate that CT can be used to detect certain characteristic manifestations in the lung associated with COVID-19.

- **Purpose**

  CT is an effective way for diagnoses, but COVID-19 and other types of pneumonia may have certain similar imaging features, thus making it difficult to differentiable. Artificial intelligence and deep learning methods are well suited to detect patterns in complex data that may be overlooked by human eye. So we try to develop a fully automatic solution to diagnose COVID-19 and evaluate the infected lung lesions based on chest CT.

Materials & Methods

The ethics committees of the participating hospitals approve this retrospective study.

- **Dataset**

  This retrospective study collects 8667 chest CT volumes from 7988 patients acquired at more than 3 medical centers. These CT scans with multiple reconstruction kernels at the same imaging session at multiple time points are included. In this study there are slightly more female cases than male. The average age is 54 ± 18 years. The detail patients' information as listed in

Table 1. All CT scans are from more than 10 CT vendors with standard imaging protocols. And about 31.73% CT scans with a slice thickness larger than 3mm (shown in Table 2).

The COVID-19 case selection criteria include: 1) RT-PCR is positive and chest CT scan is positive; 2) First RT-PCR is negative and chest CT scan is positive (these cases are from Wuhan, there are 386 volumes in our study). Pneumonia and Non-pneumonia chest CT scans are randomly selected based on the clinical report from before May 2019.

Table 1. The patient demographic statistics

	**Gender**		**Age**	
**Category**	Female	Male	>50year	<=50year
**Patients**	3826	4162	4851	3137

Table 2. CT scans statistics

	**Thickness**		**CT Vendor**					
**Category**	<=3mm	>3mm	Siemens	Philips	UIH	GE	Toshiba	Others
**Volumes**	5453	2535	2898	411	1783	2111	675	110

Table 3. Disease type information

	**Disease type**		
**Category**	COVID-19	Pneumonia	Non-pneumonia
**Volumes**	3722	2491	2454

- **Methods & Procedure**

Our study includes two main functions: COVID-19 diagnoses and infected lung lesions evaluation based on chest CT imaging. We develop a 3D deep learning framework that is shown in Fig.1.

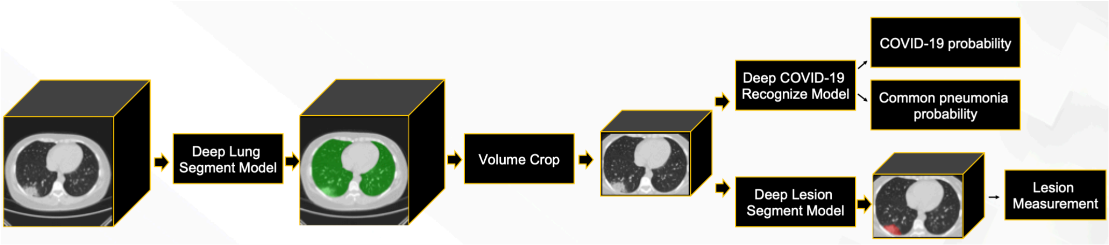

Fig 1. Workflow of COVID-19 AI assisted analysis based on chest CT imaging

The concrete steps are listed as following:

1) Step 1: Data labeling.

   - Expert radiologist labels the all datasets, and the lung region and infected lung lesions are labeled.

2) Step 2: Data pre-processing.

   - Pixel value normalization based on window level = -450, window width = 1350.

3) Step 3: Deep lung segmentation model training.
4) Step 4: Pre-processing before classification and infected lung lesion segmentation.

   - Run deep lung segmentation model.
   - Crop lung 3D region based on the lung segmentation results.
   - Resize 3D lung ROI to 256 x 256 x 64.

5) Step 5: Deep COVID-19 classification model training.
6) Step 6: Deep lung infected lesion segmentation model training.

Specifically, given a 3D chest CT scan, we first preprocess it and extract 3D lung region based deep lung segmentation model. Then the pre-processed 3D patch is passed into deep COVID-19 classification model and deep lung lesion segmentation model for predictions and lesion segmentation.

Results

This section we listed the detailed experiments information about the datasets and results.

- **Dataset**

All the chest CT volumes randomly split with a ratio of 80:20 into a training

set and testing set at the patient level. And the testing set is not used for training absolutely. The detail info is summarized in Table 4.

Table 4. Training and testing data information

	Training	Testing	Sum
**COVID-19**	3067	655	3722
**Pneumonia**	1996	495	2491
**Non-pneumonia**	1975	479	2454
**Sum**	7038	1629	8667

* Note: In order to standardize quantization, we randomly selected 400, 400, 400 covid-19, pneumonia and non-pneumonia cases from all testing data for testing.

- **Model Performance**

1) Performance of deep COVID-19 classification model

The performance of deep COVID-19 classification model is summarized in Table 5. The sensitivity and specificity for COVID-19 are 0.985 and 0.992 on the test dataset (1200 volumes) respectively.

Table 5. The performance of deep COVID-19 classification model on test set.

	Accuracy	F1-Score	Precision	Recall	Specificity
**COVID-19**	0.990	0.985	0.985	0.985	0.992
**Pneumonia**	0.965	0.947	0.968	0.927	0.985
**C&P**	0.973	0.959	0.940	0.980	0.980

*C&P: COVID-19 & Pneumonia

Fig 2 shows a representative example of COVID-19 case (RT-PCR confirmed) that is misclassified as pneumonia. The slice thickness of this case is 2mm, but only a few consecutive slices have infected small lung lesions (red arrow).

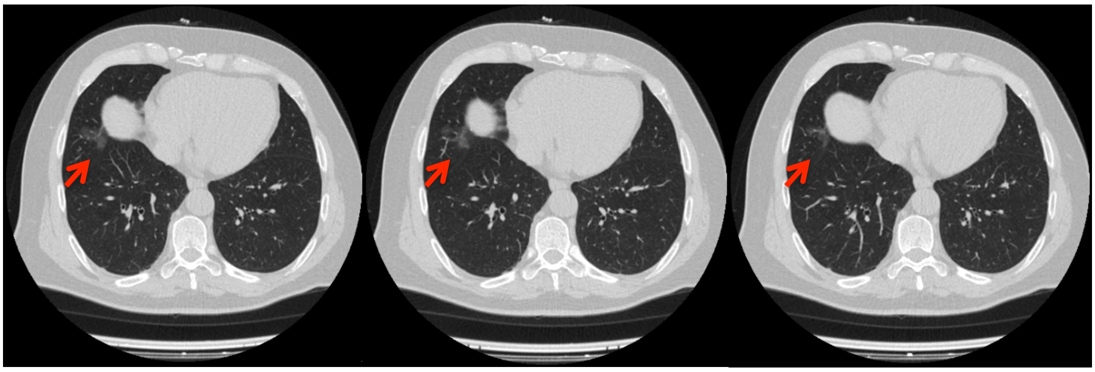

Fig 2. An example of COVID-19 case that is misclassified as pneumonia.

2) Performance of deep lung lesion segmentation model

The average DICE index on testing set is 0.75.

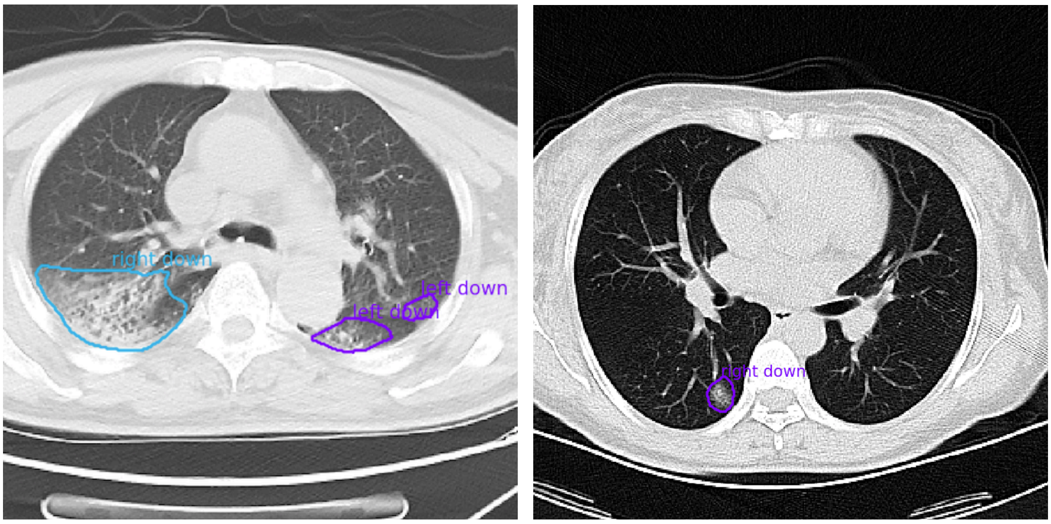

Fig 3. Segmentation results of infected lung legions from deep model.

Conclusion & Discussion

In this study, we design and evaluate a COVID-19 AI assisted analysis solution based on chest CT imaging. And the testing result demonstrates that this solution achieves high sensitivity and specificity in COVID-19 diagnosis and output the infected lung lesion at the same time. But this study only focuses on the COVID-19 diagnosis and lesion measurement. In future, we will try to address on the severity degree of COVID-19 based on the lesions features.

Experiment Data： 7 May, 2020
