# Supplementary material for: Accuracy of deep learning-based computed tomography diagnostic system for COVID-19: A consecutive sampling external validation cohort study
Source: PLoS One. 2021 Nov 4;16(11):e0258760. doi: 10.1371/journal.pone.0258760 (PMC8568139; doi:10.1371/journal.pone.0258760)
Supplement: S2 File — (DOCX) [file pone.0258760.s008.docx]

Methods regarding simulation of sensitivity, specificity, and AUC considering imperfect reference of RT-PCR

First, we calculated the sensitivity of CT-AI for true COVID-19(*SeCT*) and the specificity of CT-AI for true COVID-19 (*SpCT*). We set ** for the sensitivity of RT-PCR and set *1* for the specificity of RT-PCR. Supposed prevalence as
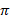

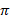
, true COVID-19 patients are and true non-COVID-19 patients are
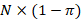
. We derived the 2*2 table as follows:

|  | | RT-PCR test | |
| --- | --- | --- | --- |
|  |  | Positive | Negative |
| CT-AI test | Positive | A | C |
|  | Negative | B | D |

*A = N*π*(Sepcr*SeCT) + N*(1-π)*((1-Sppcr)*(1-SpCT))*

*B = N*π*(Sepcr*(1-SeCT)) + N*(1-π)*((1-Sppcr)*SpCT)*

*C = N*π*((1-Sepcr)*SeCT) + N*(1-π)*(Sppcr*(1-SpCT))*

*D = N*π*((1-Sepcr)*(1-SeCT)) + N*(1-π)*(Sppcr*SpCT)*

When we substitute 1 for *Sppcr*, the following results are obtained:

*A = N*π*(Sepcr*SeCT)*

*B = N*π*(Sepcr*(1-SeCT))*

*C = N*π*((1-Sepcr)*SeCT) + N*(1-π)*(1-SpCT)*

*D = N*π*((1-Sepcr)*(1-SeCT)) + N*(1-π)*SpCT*

Suppose
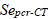
 is the sensitivity of CT-AI when RT-PCR is established as the reference, the following results are obtained:

=

Hence, the sensitivity of CT-AI when RT-PCR is established as the reference is the true sensitivity of CT-AI for COVID-19 even if the sensitivity of RT-PCR is variable.

Supposed
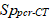
 as the specificity of CT-AI when RT-PCR is set as reference, the following results are obtained:

Now, because the specificity of RT-PCR is *1,* we can derive another 2*2 table as follows:

|  | | True Disease | |  |
| --- | --- | --- | --- | --- |
|  |  | COVID-19 | Non-COVID-19 |  |
| RT-PCR test | Positive | X | 0 | X = NPCR(+) |
|  | Negative | Y | Z | Y+Z = NPCR(-) |
|  | | X+Y | Z | X+Y+Z = N |

Supposed
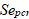
as the sensitivity of CT-AI when RT-PCR is set as reference, we derive the following:

We simulated the ROC curve of CT-AI using this information and R. The maximum is 1. We want to know the minimum . The lower , the higher the highest true specificity of CT-AI. However, the highest true specificity of CT-AI must be under 1. We used this information for R code. The R code is given below.

ROC0 <- function( disease,

normal,

lowest=NULL,

width=NULL)

{

my.hist <- function(x, brks)

{

k <- length(brks)

freq <- numeric(k)

for (i in 1:(k-1)) {

freq[i] <- sum(brks[i] <= x & x < brks[i+1])

}

freq[k] <- sum(x >= brks[k])

freq

}

x <- c(disease, normal)

min.x <- min(x)

max.x <- max(x)

if (is.null(lowest) || is.null(width)) {

temp<- pretty(c(disease, normal), n=min(length(disease)+length(normal), 50))

lowest <- temp[1]

width <- diff(temp)[1]

}

brks <- seq(lowest, max.x+width, by=width)

ROC(brks, my.hist(disease, brks), my.hist(normal, brks))

}

yoden_fun <- function(d)

{

sum = 0

best_sens = 0

best_spec = 0

best_point = 0

for (i in 1:nrow(d)){

x = d[i,1]

sens = d[i,2]

spec = d[i,3]

if((sens+spec)>sum){

sum = sens+spec

best_sens = sens

best_spec = spec

best_point = x

}

}

return(list(best_point, best_sens, best_spec))

}

# 1.1 Crude

ROC <- function( x,

disease,

normal)

{

k <- length(x)

stopifnot(k == length(disease) && k == length(normal))

Sensitivity <- c(rev(cumsum(rev(disease)))/sum(disease), 0)

False.Positive.Rate <- c(rev(cumsum(rev(normal)))/sum(normal), 0)

Sp_pcrct <- 1-False.Positive.Rate

plot(False.Positive.Rate, Sensitivity, type="b")

abline(h=c(0, 1), v=c(0, 1))

c.index <- sum(sapply(1:k, function(i)

(False.Positive.Rate[i]-False.Positive.Rate[i+1])*　(Sensitivity[i+1]+Sensitivity[i])/2))

# area under ROC curve

result <- cbind(x, disease, normal, Sensitivity[-k-1], Sp_pcrct[-k-1], False.Positive.Rate[-k-1])

rownames(result) <- as.character(1:k)

colnames(result) <- c("Value", "Disease", "Normal",

"Sensitivity", "Specificity", "F.P. rate")

d <- cbind(x, Sensitivity[-k-1], Sp_pcrct[-k-1])

d <- yoden_fun(d)

return(list(result=result, c.index=c.index, d=d))

}

disease.x <- dat %>%

dplyr::filter(PCR == 1)

normal.x <- dat %>%

dplyr::filter(PCR == 0)

ROC0(disease.x$Alibaba.Confidence, normal.x$Alibaba.Confidence)

# 1.2 Considering PCR Sensitivity

ROC <- function( x,

disease,

normal)

{

k <- length(x)

stopifnot(k == length(disease) && k == length(normal))

Sensitivity <- c(rev(cumsum(rev(disease)))/sum(disease), 0)

False.Positive.Rate <- c(rev(cumsum(rev(normal)))/sum(normal), 0)

total <- N

pos <- NPCR_ps

nega <- NPCR_ng

for (i in 1:500){

Sens_pcr <- 1 - 0.001 * i

Sp_pcrct <- 1-False.Positive.Rate

Specificity <- (nega*Sp_pcrct*Sens_pcr -pos*(1-Sens_pcr)*

(1-Sensitivity))/(total*Sens_pcr-pos)

print(i)

s = 0

for (j in 1:50){

if (Specificity[j] > s) {s <- Specificity[j]}

}

if (s >= 1) [30]

}

Sens_pcr <- Sens_pcr + 0.001

Sp_pcrct <- 1-False.Positive.Rate

Specificity <- (nega*Sp_pcrct*Sens_pcr-pos*(1-Sens_pcr)*(1-Sensitivity))

/(total*Sens_pcr-pos)

False.Positive.Rate <- 1-Specificity

plot(False.Positive.Rate, Sensitivity, type="b")

abline(h=c(0, 1), v=c(0, 1))

c.index <- sum(sapply(1:k, function(i)

(False.Positive.Rate[i]-False.Positive.Rate[i+1])*　(Sensitivity[i+1]+Sensitivity[i])/2))

# area under ROC curve

result <- cbind(x, disease, normal, Sensitivity[-k-1],

Specificity[-k-1], False.Positive.Rate[-k-1])

rownames(result) <- as.character(1:k)

colnames(result) <- c("Value", "Disease", "Normal", "Sensitivity",

"Specificity", "F.P. rate")

d <- cbind(x, Sensitivity[-k-1], Specificity[-k-1])

d <- yoden_fun(d)

return(list(result=result, c.index=c.index, d=d))

}

disease.x <- dat %>%

dplyr::filter(PCR == 1)

normal.x <- dat %>%

dplyr::filter(PCR == 0)

ROC0(disease.x$Alibaba.Confidence, normal.x$Alibaba.Confidence)
